# A Rare Case of Adult Colonic Intussusception from Benign Etiology

**DOI:** 10.7759/cureus.2023

**Published:** 2018-01-04

**Authors:** Tagore Sunkara, Megan E Caughey, Andrea Culliford, Vinaya Gaduputi

**Affiliations:** 1 Internal Medicine/gastroenterology, The Brooklyn Hospital Center, Affiliate of the Mount Sinai Hospital. 121 Dekalb Avenue, Brooklyn, Ny 11201; 2 Internal Medicine, New York Institute of Technology College of Osteopathic Medicine, Old Westbury, Ny; 3 Department of Internal Medicine, SBH Health System, 4422 Third Ave, Bronx, Ny 10457

**Keywords:** intussusception, adult intussusception, bowel obstruction

## Abstract

Defined as the tunneling of one bowel segment into an adjacent bowel segment, intussusception is typically observed in pediatric populations. Here, we present the case of a 78-year-old man who, in a series of unlikely events, developed colonic intussusception due to a benign lead point pathology. Intussusception of the colon is an uncommon occurrence in adults. However, adult colonic intussusception, observed in the absence of a malignant lead point pathology, represents a true clinical anomaly.

## Introduction

Intussusception of the bowel is one of the most common abdominal emergencies affecting those under two years of age. It is an appreciably more rare finding in adult populations compared to pediatric ones. When it does arise in adults, it tends to involve the small bowel more often than the colon. Furthermore, when colonic intussusception occurs in an adult, it is usually due to a malignant lead point pathology, like primary adenocarcinoma in particular [1]. Thus, intussusception presenting in an adult rather than a child, in the colon rather than the small bowel, and from a benign rather than a malignant etiology, is unusual from numerous perspectives.

## Case presentation

A 78-year-old man, with a medical history of hypertension, diabetes mellitus II, hyperlipidemia, an implanted loop recorder, legal blindness, and residual left-sided weakness resulting from a cerebrovascular accident, was bought into the emergency department complaining of abdominal pain for one week. The patient complained of chronic constipation and left lower quadrant pain for one year, with exacerbating symptoms the past week. The pain was described as five on a scale of 10 on pain scale, intermittent, sharp, and worse with eating or straining. He had also noted increasing abdominal distension, generalized weakness, and two episodes of non-bilious, non-bloody vomiting for four days prior to coming to the hospital. After taking a fleet enema the day before, the patient had produced a large volume of watery, non-bloody stool. He admitted to a twenty-pound unintentional weight loss but was unable to specify the period of time over which that weight loss had occurred. In an interview, the patient denied any prior abdominal surgeries. The patient also claimed that he had three screening colonoscopies done in his life time, the last one at the age of 72. All of the colonoscopies were normal.

On presentation, his vitals were stable. On physical exam, the abdomen was distended and mildly tense, with tenderness in both lower quadrants. On auscultation, the bowel sounds were diminished. No abdominal wall hernia was noted. A computed tomography (CT) scan of the abdomen showed dilated small bowel loops with air-fluid levels and a distended large bowel. Also found was evidence of right transverse and left colon diffuse wall thickening and mucosal irregularity, a constricting mass-like lesion in the proximal sigmoid colon, with significant narrowing, and diverticulosis. There were no signs of bowel ischemia noted on the CT scan. The patient was subsequently put nil per os (NPO) and given nasogastric suction, intravenous fluids, and intravenous ciprofloxacin and metronidazole.

**Figure 1:**
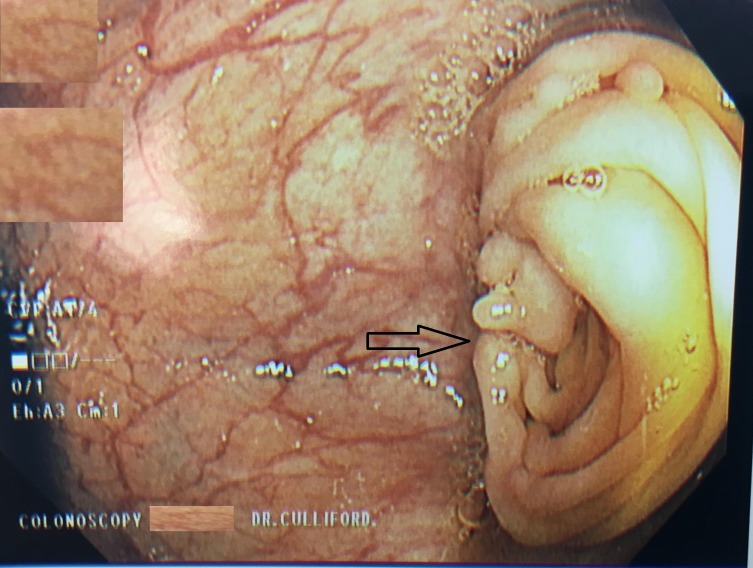
Colonoscopy revealing colonic intussusception at 30 cm from the anal verge

A colonoscopy was performed, and at 30 cm, an intussusception of the colonic mucosa was identified (Figure 1).

The scope was unable to pass beyond this point, and the patient developed bradycardia, with a heart rate around 30 per minute, so air was removed and the procedure was aborted. Colorectal surgery was consulted and an exploratory laparotomy with mobilization of the splenic flexure was performed. Resection of the sigmoid colon mass along with the adherent ileum and peritoneum was performed. The patient was also given a small bowel side-to-side anastomosis and an end-to-side large bowel anastomosis. A total of 40 cm colon was resected during the surgery. The pathology report of the excised mass showed chronic serosal inflammation and mild fibrosis and determined that the mass was ultimately negative for malignancy. The patient's postoperative care was uneventful and he was discharged home one week after the procedure. The patient was followed up one month after discharge at which point he denied any complaints. The patient is also scheduled to follow up with colorectal surgery.

## Discussion

Adult bowel intussusception is a rare clinical finding, accounting for only 5% of all instances of intussusception. Although it commonly first manifests as an obstructive process, only 1-5% of all bowel obstructions are due to adult intussusception. Adult bowel intussusceptions are diagnostically challenging because they can present only with vague abdominal discomfort without any typical obstructive symptoms [2].The patient we describe here serves as confirmation, for he presented with constipation and intensifying abdominal discomfort in the absence of vomiting and hematochezia.

It is estimated that half of all adult bowel intussusceptions result from malignancy, and for this reason, treatment with surgical resection is often unavoidable [3]. One study that examined 43 total instances of intussusception over a 10-year period of time, found that 67% of intussusceptions involved a lead point. This same study discovered that CT was the preferred diagnostic imaging modality (81%), and that surgical intervention was required in the majority of cases (72%) [4]. The case we describe here is particularly unique because in addition to being an example of intussusception in an adult, it occurred in the colon and was not associated with a malignant lesion. This represents a true outlier because adult colonic intussusceptions are rare to begin, and it has been noted that up to 58% of colonic intussusceptions are due to malignant and not benign tumors [5].

Barium enema examinations, endoscopy, and abdominal CT imaging are useful diagnostic tools in identifying patients with colonic intussusception. However, CT is widely regarded as the key to diagnosis, for it can quickly identify intussusception, ensuring appropriate intervention in a timely manner [6]. In particular, through CT imaging, it is possible to differentiate intussusception due to a lead point from intussusception occurring in the absence of one. This has important implications for avoiding unnecessary surgical measures. If an intussusception has a lead point, the CT usually reveals signs of bowel obstruction, a lead mass, or edema disrupting the three-layer appearance of the bowel. The patient in this case report had a lead mass, colon wall thickness irregularities, and evidence of bowel obstruction [7].

In a case similar to the one we describe here, a 47-year-old woman developed colonic intussusception in the descending colon as a result of a colonic lipoma. She also cited experiencing constipation over the past four years and abdominal pain or distension in the absence of other symptoms. Although a lipoma is a benign adipose tumor, after it was identified on CT, it was removed through a laparoscopic resection [8]. In another instance, a 31-year-old woman was found to have ascending colonic intussusception following a colonoscopy. After an ileocolic resection was performed, it was determined that the colonic intussusception was not due to any underlying pathology. It is unusual for colonic intussusception to occur due to a benign lesion or after a more minor procedure [9].

In adults, intussusception is managed less conservatively than it is managed in children. Most children can be treated non-surgically first, with either air, water-soluble, or saline enemas. This is especially true at institutions with pediatric surgery departments to rely on for backup support in the case of initial treatment failure. In adults, because malignancy is such a concern, this treatment option is often limited to instances of transient non-obstructive intussusception. It is estimated that 70-90% of adults with intussusception, undergo surgical resection, much like the patient we presented here [10].

## Conclusions

Adult colonic intussuseption is a very rare entity and it is even more unusual for it to be a result of a benign etiology. A clinician must be acutely aware of this condition, and when the condition is suspected, diagnosis should be made promptly, failing which serious complications such as bowel obstruction, necrosis, and perforation can occur. Definitive treatment in adults is often surgical in contrast to pediatric cases where conservative management resolves the condtion in a majority of patients.

## References

[1] Azar T, Berger DL (1997). Adult intussusception. Ann Surg.

[2] Wilson A, Elias G, Dupiton R (2013). Adult colocolic intussusception and literature review. Case Rep Gastroenterol.

[3] Rogy M, Mirza D, Berlakovich G, Winkelbauer F, Rauhs R (1991). Submucous large-bowel lipomas—presentation and management. An 18-year study. Eur J Surg.

[4] de Clerck F, Vanderstraeten E, De Vos M, Van Steenkiste C (2016). Adult intussusception: 10-year experience in two Belgian centres. Acta Gastroenterol Belig.

[5] Barussaud M, Regenet N, Briennon X (2006). Clinical spectrum and surgical approach of adult intussusceptions: a multicentric study. Int J Colorectal Dis.

[6] Zerwas E, Kemper-Martin A, Comes A (2008). A case report of adult colonic intussusception. J La State Med Soc.

[7] Loukas M, Pellerin M, Kimball Z, de la Garza-Jordan J, Tubbs RS, Jordan R (2011). Intussusception: an anatomical perspective with review of the literature. Clin Anat.

[8] Saba RB, Sadeghi A, Rad N, Safari MT, Barzegar F (2016). Colonic intussusception in descending colon: an unusual presentation of colon lipoma. Gastroenterol Hepatol Bed Bench.

[9] Min MX, Sklow B, Vaughn BP (2017). Intussusception after routine colonoscopy: a rare complication. ACG Case Rep J.

[10] Khan Z, Darr U, Renno A, Alkully T, Rafiq E, Sodeman T (2017). Transient descending colonic intussusception due to a large fecaloma in an adult. ACG Case Rep J.

